# Limited emergence of resistance to integrase strand transfer inhibitors (INSTIs) in ART-experienced participants failing dolutegravir-based antiretroviral therapy: a cross-sectional analysis of a Northeast Nigerian cohort

**DOI:** 10.1093/jac/dkad195

**Published:** 2023-06-27

**Authors:** Adam Abdullahi, Ibrahim Musa Kida, Umar Abdullahi Maina, Amina Husaini Ibrahim, James Mshelia, Haruna Wisso, Abdullahi Adamu, James Ezenwa Onyemata, Martin Edun, Haruna Yusuph, Sani H Aliyu, Man Charurat, Alash’le Abimiku, Lucie Abeler-Dorner, Christophe Fraser, David Bonsall, PANGEA consortium, Lucie Abeler-Dörner, Helen Ayles, David Bonsall, Rory Bowden, Vincent Calvez, Max Essex, Sarah Fidler, Christophe Fraser, Kate Grabowski, Tanya Golubchik, Ravindra Gupta, Richard Hayes, Joshua Herbeck, Joseph Kagaayi, Pontiano Kaleebu, Jairam Lingappa, Sikhulile Moyo, Vladimir Novitsky, Thumbi Ndung'u, Deenan Pillay, Thomas Quinn, Andrew Rambaut, Oliver Ratmann, Janet Seeley, Deogratius Ssemwanga, Frank Tanser, Maria Wawer, Myron Cohen, Tulio D'Oliveira, Ann Dennis, Max Essex, Sarah Fidler, Dan Frampton, Christophe Fraser, Tanya Golubchik, Richard Hayes, Josh Herbeck, Anne Hoppe, Pontiano Kaleebu, Paul Kellam, Cissy Kityo, Andrew Leigh-Brown, Jairam Lingappa, Vladimir Novitsky, Nick Paton, Deenan Pillay, Tom Quinn, Oliver Ratmann, Deogratius Ssemwanga, Frank Tanser, Maria Wawer, Steven A Kemp, Ravindra K Gupta

**Affiliations:** Department of Medicine, Cambridge Institute of Therapeutic Immunology & Infectious Disease (CITIID), Cambridge, UK; Department of Medicine, University of Cambridge, Cambridge, UK; Institute of Human Virology Nigeria, Abuja, Nigeria; Department of Infectious Disease and Clinical Immunology, University of Maiduguri, Borno, Nigeria; Department of Veterinary Pharmacology and Toxicology, Faculty of Veterinary Medicine, University of Maiduguri, Borno, Nigeria; Federal Medical Centre, Abuja, Nigeria; Department of Infectious Disease and Clinical Immunology, University of Maiduguri, Borno, Nigeria; Institute of Human Virology Nigeria, Abuja, Nigeria; Department of Veterinary Pharmacology and Toxicology, Faculty of Veterinary Medicine, University of Maiduguri, Borno, Nigeria; Institute of Human Virology Nigeria, Abuja, Nigeria; Institute of Human Virology Nigeria, Abuja, Nigeria; Department of Infectious Disease and Clinical Immunology, University of Maiduguri, Borno, Nigeria; Department of Microbiology, Addenbrooke’s Hospital, Cambridge University Hospitals NHS Foundation Trust, Cambridge, UK; Institute of Human Virology, University of Maryland School of Medicine, Baltimore, USA; Institute of Human Virology Nigeria, Abuja, Nigeria; Nuffield Department of Medicine, Big Data Institute, University of Oxford, Oxford, UK; Nuffield Department of Medicine, Big Data Institute, University of Oxford, Oxford, UK; Nuffield Department of Medicine, Big Data Institute, University of Oxford, Oxford, UK; Department of Medicine, Cambridge Institute of Therapeutic Immunology & Infectious Disease (CITIID), Cambridge, UK; Department of Medicine, University of Cambridge, Cambridge, UK; Nuffield Department of Medicine, Big Data Institute, University of Oxford, Oxford, UK; Department of Medicine, Cambridge Institute of Therapeutic Immunology & Infectious Disease (CITIID), Cambridge, UK; Department of Medicine, University of Cambridge, Cambridge, UK; Africa Health Research Institute, Durban, South Africa

## Abstract

**Background:**

Due to the high prevalence of resistance to NNRTI-based ART since 2018, consolidated recommendations from the WHO have indicated dolutegravir as the preferred drug of choice for HIV treatment globally. There is a paucity of resistance outcome data from HIV-1 non-B subtypes circulating across West Africa.

**Aims:**

We characterized the mutational profiles of persons living with HIV from a cross-sectional cohort in North-East Nigeria failing a dolutegravir-based ART regimen.

**Methods:**

WGS of plasma samples collected from 61 HIV-1-infected participants following virological failure of dolutegravir-based ART were sequenced using the Illumina platform. Sequencing was successfully completed for samples from 55 participants. Following quality control, 33 full genomes were analysed from participants with a median age of 40 years and median time on ART of 9 years. HIV-1 subtyping was performed using SNAPPy.

**Results:**

Most participants had mutational profiles reflective of exposure to previous first- and second-line ART regimens comprised NRTIs and NNRTIs. More than half of participants had one or more drug resistance-associated mutations (DRMs) affecting susceptibility to NRTIs (17/33; 52%) and NNRTIs (24/33; 73%). Almost a quarter of participants (8/33; 24.4%) had one or more DRMs affecting tenofovir susceptibility. Only one participant, infected with HIV-1 subtype G, had evidence of DRMs affecting dolutegravir susceptibility—this was characterized by the T66A, G118R, E138K and R263K mutations.

**Conclusions:**

This study found a low prevalence of resistance to dolutegravir; the data are therefore supportive of the continual rollout of dolutegravir as the primary first-line regimen for ART-naive participants and the preferred switch to second-line ART across the region. However, population-level, longer-term data collection on dolutegravir outcomes are required to further guide implementation and policy action across the region.

## Introduction

Due to the increasing prevalence of pre-treatment NNRTI drug resistance,^1–3^ the WHO has (since 2018) recommended the use of dolutegravir as the preferred ART drug of choice for both newly diagnosed and individuals transitioning from previous regimens.^4^ This decision was supported by numerous safety, potency, tolerability and cost-effective characteristics of dolutegravir.^5^ Since 2019, more than 50 countries across sub-Saharan Africa (SSA) have rolled out (or have plans to roll out) dolutegravir as part of standard treatment. The continuing rollout is aided by the availability of a low-cost, generic fixed-dose co-formulation of tenofovir disoproxil fumarate, lamivudine and dolutegravir, called TLD.^6^

Dolutegravir-based ART has been commercialized and distributed in Nigeria since late 2019. As of 2020, national Nigerian treatment guidelines recommend the transition to dolutegravir-based ART in both virally suppressed and unsuppressed participants.^7^ However, due to economic and other factors, there is no routine virological or resistance testing in Nigeria. Therefore, the majority of HIV-1-infected participants transitioned to dolutegravir-based regimens without prior viral load (VL) or resistance testing. Data from the ADVANCE and NAMSAL clinical trials,^8,9^ which recruited ART-naive participants exclusively in SSA, showed no evidence of emergence of drug resistance-associated mutations (DRMs) amongst participants on dolutegravir-based ART. Data on treatment outcomes of ART-experienced participants transitioning to TLD are limited, though some literature is beginning to emerge.^10^ Despite this, there are almost no data available from West Africa.

Given the high prevalence of ART resistance amongst treatment-experienced, HIV-1 participants following virological failure whilst on previous first- and second-line ART regimens,^11^ data on resistance outcomes following long-term use of dolutegravir are highly valuable. In this study, we present resistance outcomes and drug-resistance genotypes using next-generation HIV-1 sequencing, from participants in a small, Nigerian, treatment-experienced cohort failing dolutegravir-based ART following rollout.

## Methods

### Study population and design

This was a cross-sectional study performed at the University of Maiduguri Teaching Hospital, Borno State, Nigeria, between January and June 2021. Assessing virological and resistance outcomes in this region of Nigeria has been challenging due to ongoing and long-term armed conflict caused by the Boko Haram insurgency, which has turned the area into a conflict zone. Inclusion criteria for this study was as follows: participants with virological failure (two consecutive HIV-1 RNA VL >1000 copies/mL) whilst following a dolutegravir-based ART regimen for >6 months, ≥18 years of age, attending routine clinic visits and able to provide informed consent for participation in the study. All participants included in this study provided informed consent prior to sample and data collection. Available demographic data including age, gender, ART regimen, duration on ART and current CD4 count were collected from clinical files and recorded in Microsoft Excel (Office 365, Microsoft, Redmond, USA).

### Laboratory methods

Plasma was separated from whole venous blood in EDTA within 2 h of collection and stored immediately at −80°C. Plasma VL testing and CD4+ counts were performed at the Defence Reference Laboratory, Asokoro Abuja using the COBAS AmpliPrep/COBAS TaqMan HIV type 1 (HIV1) v2.0 test (Roche Diagnostics, Basel, Switzerland). WGS was performed retrospectively using the ve-SEQ-HIV^12^ protocol on 61 plasma samples with VL > 1000 copies/mL and stored prior to confirmation of VL.

Briefly, total RNA was extracted from plasma samples, washed in ethanol, and eluted using the NUCLISENS easyMAG system (bioMérieux). Libraries were prepared using the SMARTer Stranded Total RNA-Seq Kits v2 (Clontech, Takara Bio) according to the manufacturer’s protocol. Total RNA was denatured, and reverse-transcribed to cDNA and a total of 500 ng of pooled libraries were hybridized to custom HIV-specific biotinylated 120-mer oligonucleotides (xGen Lockdown Probes, Integrated DNA Technologies). Captured libraries were then amplified by PCR to produce a final pool for sequencing with an Illumina MiSeq (San Diego, CA, USA) to produce up to 300-nucletoide paired-end reads.

### Bioinformatics analysis

FastQ files were trimmed of all sequencing adapters, and mapped iteratively to the best available reference sequence, from a curated alignment of 3000 HIV-1 genomes downloaded from the Los Alamos HIV sequence database, using SHIVER.^13,14^ BAM files were quality controlled by determining the read depth at each position of the HIV-1 genome. Sequences with a depth of <50 reads were excluded from further analysis. Resistance genotyping was performed using an in-house script that utilizes the Stanford HIV drug resistance algorithm (v9.1) but is adapted to calculate the prevalence of DRMs from all available sequencing data rather than just consensus-level mutations. The script calls mutations with an absolute minimum read count of 50 reads, and minimum frequency of 2%, 10% and 20%. Haplotypes were reconstructed using CliqueSNV v2.0.3^15^ using the -fdf extended, -m snv-illumina and -tf 0.05 flags. Phylogenies were inferred with IQ-TREE2 v2.2.2^15^ using a GTR + F + I + R4 model with 1000 rapid bootstraps. Inference of transmission was made with phyloscanner v1.8.1^13^ using overlapping windows of 150 bp across the whole genome. Phylogenies were rooted on an HIV-1 subtype G reference sequence, downloaded from the curated HIV-1 alignment from the Los Alamos National HIV Database. HIV-1 subtyping was performed using SNAPPy v1.0.0.^16^ Prediction of co-receptor usage was made using TROPHIX (prediction of HIV-1 tropism; http://sourceforge.net/projects/trophix/).

### Ethics

The study was approved by the University of Maiduguri Teaching Hospital Ethics Committee (UMTH/REC/21/714). All participants provided written informed consent.

## Results

**Figure 1.**
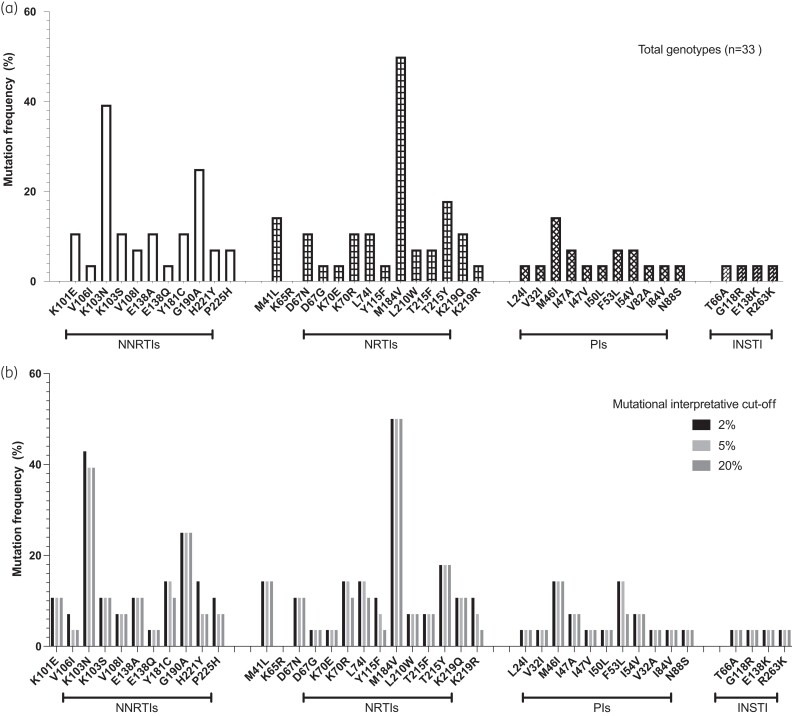
(a) Proportion of participants with DRMs using the Stanford algorithm. (b) Proportion of participants with DRMs using the Stanford algorithm, subdivided into interpretational cut-offs of 2%, 5% and 20%. Evidence suggests that minority variants may play a role in drug resistance.^21^

Virological assessment revealed that of 4263 HIV-1-positive participants on dolutegravir-based ART for ≥6 months, 452 (11%) had a detectable VL (>400 copies/mL), yielding a population-level suppression rate of 89%. Amongst those participants with a detectable VL, 281 had a VL of >1000 copies/mL; plasma collection was successful in 61 participants. Following quality control of the WGS, due to poor coverage and depth across most of the genome (Figure 1a and b, available as Supplementary data at *JAC* Online), 28 samples were excluded from further analysis. This resulted in a population of 33 participants with a fully intact whole genome, from which the prevalence of DRMs and minority variants were calculated.

This population (Table 1) was a largely ART-experienced cohort, with almost two-thirds switching from PI-based second-line ART, to dolutegravir-based ART. The median duration of ART was 9 years, with a median CD4+ count of 200 cells/mm^3^, and a median VL of 4.1 log_10_ copies/mL (range 3.0–5.2). Only two participants were initiating first-line dolutegravir-based regimens; the remainder were switched to dolutegravir as their second-line regimen. We did not observe from clinical records, any previous exposure to an INSTI in our study population.

**Table 1.** Characteristics of study participants with successful WGS and genotyping^a^

Characteristic	
Participants	
* *Total number of participants	33
* *Female, *n* (%)	20 (61)
* *Age, years, median (IQR)	40 (35–48)
* *CD4+ count, cells/mm^3^, median (IQR)	200 (300–467)
Current ART regimen	
* *TDF + 3TC + DTG, *n* (%)	33 (100)
* *Time on DTG, years, median (IQR)	1.8 (1.4–1.9)
* *Time on ART, years, median (IQR)	9.3 (5.8–15.0)
* *Switching to DTG, *n* (%)	31 (94)
* *Starting DTG, *n* (%)	2 (6)
ART regimen prior to DTG, *n*(%)	
* *TDF + 3TC + LPV/r	17 (52)
* *TDF + 3TC + ATV/r	4 (12)
* *ZDV + 3TC + EFV	8 (24)
* *ABC + 3TC + EFV	2 (6)
* *No prior ART	2 (6)
HIV-1 subtype, *n* (%)^b^	
* *G	13 (39)
* *G/A1	5 (15)
* *CRF02_AG	3 (9)
* *A (A1)	2 (6)
* *CRF02_AG/G	2 (6)
* *02AG/A1	2 (6)
* *Others	6 (18)

TDF, tenofovir disoproxil fumarate; 3TC, lamivudine; DTG, dolutegravir; LPV, lopinavir; r ritonavir; ATV, atazanavir; ZDV, zidovudine; EFV, efavirenz; ABC, abacavir.

Data available from 33 participants following quality control.

Other subtypes comprising: CRF06_CPX (*n* = 1); CRF13_CPX-like (*n* = 1); CRF13_CPX/G (*n* = 1); CRF 11_CPX (*n* = 1); G/C (*n* = 1); G/J/A (*n* = 1).

Using the SNAPPy HIV-1 subtyping tool, ∼40% of viruses were assigned to subtype G, and 15% were G_A1 subtypes. The remaining viruses were recombinants, as expected from previous work (Table 1).^17,18^ Using a minimum threshold of 20%, NRTI, NNRTI, PI and INSTI DRMS occurred in 17 (52%), 24 (73%), 4 (12%) and 1 (3%) of samples, respectively (Figure 1a). Dual-class resistance occurred in 17 (50%) participants and tri-class mutations occurred in 5 (14.7%) participants, which is consistent with long-term exposure to lamivudine. The most prevalent NRTI mutation was M184V,^19^ though we also observed occurrence of L74I in two cases (6%); this mutation compensates for the fitness defect induced by M184V.^20^ The most prevalent NNRTI DRM was K103N, reflective of prior exposure to nevirapine and efavirenz in this cohort of treatment-experienced persons living with HIV (PLWH). The frequency of detected DRMs was similar across the different interpretive thresholds of 2%, 10% and 20% (Figure 1b).

One participant, aged 18 years, known to be vertically infected, was found to have high-level resistance to NRTIs, NNRTIs and INSTIs, including almost complete resistance to the long-acting injectable cabotegravir. Mutations included inE138K, inG118R, inT66A, inR263K, rtH221Y, rtV108I, rtK103N, rtM184V, rtM41L, rtA98G and rtT215Y, all at frequencies >40%, with a mean read depth of 770×. This participant was established on dolutegravir for a median of 1.2 years and on ART for a median of 12 years. Data on other clinical parameters including nadir CD4+ counts were unavailable.

**Figure 2.**
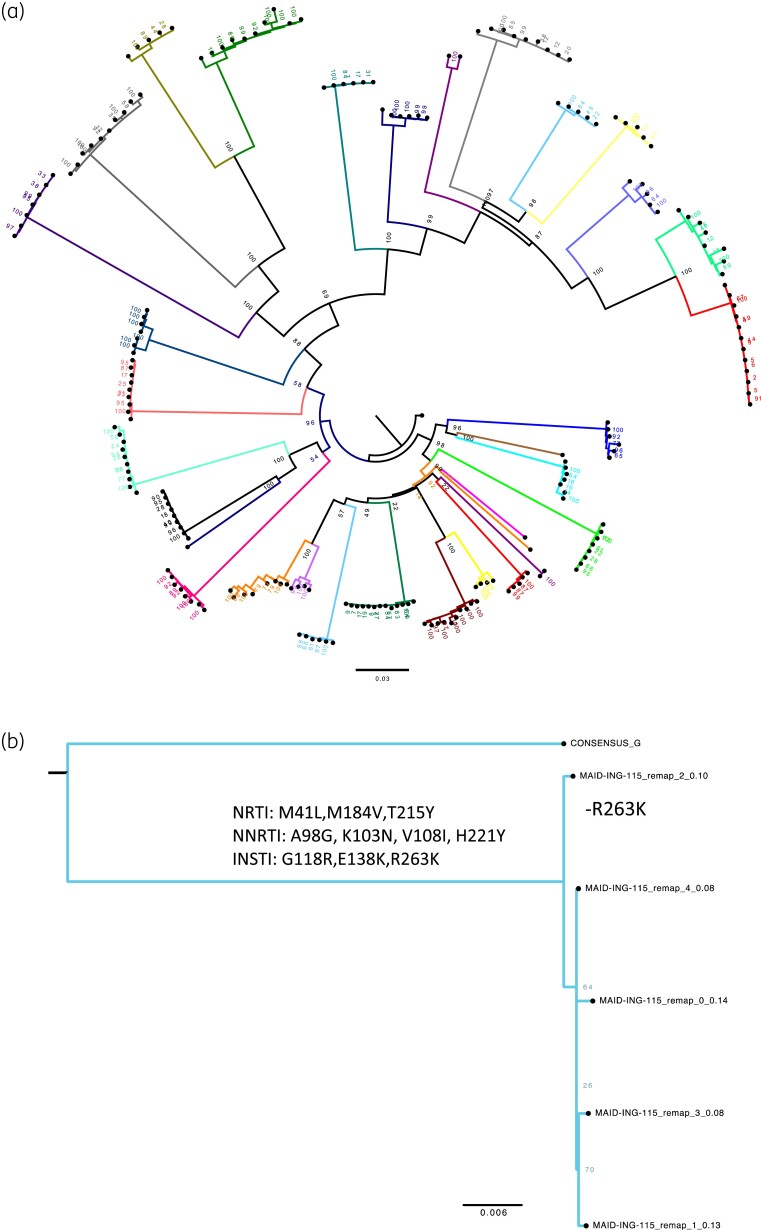
(a) Maximum-likelihood phylogeny of reconstructed haplotypes for all participants and (b) only the patient with cabotegravir resistance, with 1000 ultrafast bootstraps (indicated at each node). Mutations defining each haplotype are listed at the node. Haplotypes were homogeneous, with little diversity or changes in frequency of DRMs. This figure appears in colour in the online version of *JAC* and in black and white in the print version of *JAC*.

To identify within-host diversity and potential transmission of DRMs between participants, we reconstructed viral haplotypes (Figure 2a) for each participant, and individually for the cabotegravir-resistant participant (Figure 2b). Viral haplotypes were largely homogeneous within host, each containing highly similar DRMs within each of the reconstructed haplotypes for each participant. Following this, we investigated whether there was evidence of transmission or genetic linkage between participants in this cohort (Figure 2). However, no significant transmission or strong degree of genetic linkages were identified.

Two participants were identified as harbouring CXCR-4 co-receptor using viruses. One of these participants had DRMs associated with reduced susceptibility to NNRTIs, characterized by K103N and E138A. The other participant had DRMs reducing susceptibility to both NRTIs and NNRTIs, characterized by M184V and G190A. Neither participant had any PI or INSTI DRMs.

## Discussion

Following the initial rollout of previous first-line, NNRTI-based ART regimens, DRMs were first observed within the first year of virological failure in ∼15%–35% of participants, characterized by resistance to lamivudine, tenofovir disoproxil fumarate and NNRTIs.^22,23^ Drug resistance is associated with increased mortality in hospitalized individuals in low-middle income (LMIC) settings.^24^ The second-generation INSTI dolutegravir has been systematically adopted and rolled out across SSA since 2018, though there are concerns regarding the emergence of DRMs following virological failure in these limited-monitoring settings. In a recent analysis of pooled evidence on virological and resistance outcomes following dolutegravir failure in SSA, there was an overall high rate of virological response to dolutegravir: 88.5% (95% CI: 73.8–97.8). Participants in these studies experienced an exceptionally low rate of virological failure,^10^ and there was minimal evidence of the emergence of DRMs during short observation periods. However, prolonged virological failure is expected to eventually lead to the selection of DRMs within viral quasispecies, as a result of intrahost evolution.^21,25^

It is worth noting that the presence of pre-existing DRMs, prior to switching to dolutegravir-based ART regimens may have a significant impact on both virological and resistance outcomes. Previous studies suggest that NNRTI mutations acquired before treatment can reduce the effectiveness of dolutegravir-based ART regimens.^26^ However, other studies conducted in various countries in SSA have reported similar virological and resistance outcomes in both ART-naive^27,28^ and ART-experienced participants^29,30^ (without evidence of DRMs), as well as in ART-experienced individuals with a history of NRTI mutations, particularly M184V/I.^31–33^ Nonetheless, it is important to acknowledge that resuppression can occur after a viral rebound, but DRMs accumulated during that rebound can lead to high-level resistance against second-line ART components.^34^

In this study, we conducted a cross-sectional analysis to determine the prevalence of DRMs, using WGS, in a small cohort of HIV-1-positive participants who were experiencing virological failure on dolutegravir-based ART regimens (defined as a VL > 1000 copies/mL). This study is particularly relevant as the majority of HIV-1 in Nigeria is subtype G or AG recombinants, rather than the commonly investigated subtype C from many other parts of SSA. Many of the viraemic participants were ART-experienced, having previously been on PI-based regimens before being switched to TLD. We attribute the high rate of virological failure amongst these participants to poor adherence. Despite this, we observed a low incidence of individuals with TDF or dolutegravir resistance despite experiencing viraemia on TLD. This is reassuring as it suggests that adherence counselling would likely result in resuppression of viraemic participants, similar to those rates observed with PI-based ART.^35^

Amongst the 33 participants with sequencing data of sufficient quality, the observed mutational landscape was reflective of exposure to previous first-line NNRTI-based regimens, with only a single participant having DRMs reducing the efficacy of dolutegravir or other PIs. This participant was vertically infected, likely at a young age, due to the length of ART treatment. The participant had a number of DRMs conferring high-level resistance to dolutegravir and other INSTIs, i.e. T66A, G118R, E138K (accessory) and R263K. The T66A mutation is non-polymorphic and primarily selected by elvitegravir and raltegravir, resulting in an ∼9-fold reduction in susceptibility to elvitegravir but minimal impact on other INSTIs. The E138K mutation has negligible effect on susceptibility to INSTIs; however, when found in combination with other DRMs, this results in further decreased susceptibility to dolutegravir.^36^ Further, the G118R and R263K mutations observed in this patient, conferring between 2- and 15-fold reductions to dolutegravir susceptibility,^37,38^ have also been observed in participants experiencing virological failure on INSTI-based regimens in non-B HIV subtypes^39–42^ and is known to reduce long terminal repeat DNA binding, replication capacity, infectivity and viral fitness.^43^ It is likely that the G118R mutation emerged first and led to the accumulation of other mutations including the E138K compensatory mutation, as G118R has been described as a dolutegravir-resistance pathway in non-B subtypes.^37,44^ Research on non-B subtype drug resistance is therefore important, especially as polymorphisms in the integrase gene may have a negative effect on impact on resistance outcomes to other INSTIs.^45^

We note that there was a complete absence of the NRTI mutation K65R, along with low-level resistance to tenofovir disoproxil fumarate. This phenomenon has also been described in other Nigerian cohorts.^46,47^ To ensure that this was a true finding, investigation of read coverage shows that there was sufficient depth to determine DRMs at this site, though there was a complete absence of it (Figure 2). We note the frequent presence of several thymidine analogue mutations (TAMs) alongside other NNRTI mutations likely reflects prior use of zidovudine, stavudine + NNRTI-based ART, prior to virological failure, with subsequent switch to tenofovir disoproxil fumarate-containing PI-based regimens. It should be noted that K65R is antagonistic to TAMs on the same genome,^21,48^ and that the occurrence of NRTI and PI mutations is relatively uncommon during viraemia on PI-based ART.^49,50^

Given the increasing use of dolutegravir in clinical practice, the most effective strategy for managing participants with persistent viraemia whilst on dolutegravir-based ART regimens remains uncertain. This is especially true in resource-limited settings such as Nigeria, where the capacity for drug resistance testing is limited.^51^ Despite this, this study has provided additional evidence to support favourable outcomes when providing long-term ART services in areas of conflict and civil unrest. However, several factors may increase the risk of dolutegravir resistance emerging in the region, including prolonged virological failure due to the lack of routine virological monitoring^52,53^ as well as suboptimal treatment adherence,^54^ which is an independent determinant of virological outcomes in these settings.

Disengagement from care is also a significant problem, although it is possible to re-engage with a substantial proportion of cases,^55^ highlighting the benefits that can be gained through outreach and tracing efforts. Given that INSTI-based long-acting injectables are being considered as pre-exposure prophylaxis,^56^ it is crucial to conduct further analyses of resistance across SSA over extended periods and to monitor newly diagnosed individuals for INSTI resistance.

This study was subject to limitations. Study sample size was small, with only 33 samples generating high-quality sequences for analysis despite sample collection from 61 participants. We speculate this could be due to human-related factors, which may include shipping and storage conditions. We did not collect data on adherence although the mutational landscape and profile of patients are consistent with prior treatment with NNRTI-based first-line regimens and with evidence of virological failure on dolutegravir. Nonetheless, given the paucity of data across this region, this study provides critical outcome and resistance data including highlighting the need for larger and more comprehensive population-level cohort studies to further understand the emerging trends and prevalence of DRMs in individuals failing dolutegravir-based ART in resource-limited settings.

Finally, given the potential for dolutegravir resistance to emerge due to suboptimal treatment adherence and prolonged virological failure, efforts to improve adherence and increase access to routine virological monitoring should be prioritized to minimize the emergence and spread of drug resistance. Further research is needed to evaluate the effectiveness of different strategies for managing individuals experiencing persistent viraemia on dolutegravir-based ART in resource-limited settings, particularly in the context of ongoing conflict and civil unrest.

## Supplementary Material

dkad195_Supplementary_Data

## Supplementary data

Figures S1 and S2 are available as Supplementary data at *JAC* Online.
