# Supplementary material for: Limited emergence of resistance to integrase strand transfer inhibitors (INSTIs) in ART-experienced participants failing dolutegravir-based antiretroviral therapy: a cross-sectional analysis of a Northeast Nigerian cohort
Source: J Antimicrob Chemother. 2023 Jun 27;78(8):2000–7. doi: 10.1093/jac/dkad195 (PMC10393879; doi:10.1093/jac/dkad195)
Supplement: dkad195_Supplementary_Data [file dkad195_supplementary_data.zip › PANGEA 1 and 2 SC.docx]

# Appendix 1: PANGEA 2 Steering Committee

| Name | Institution | Representatives & roles | Email address^1^ |
| --- | --- | --- | --- |
| Lucie Abeler-Dörner | University of Oxford | Project manager | lucie.abeler-dorner@bdi.ox.ac.uk |
| Helen Ayles | PopART/ Zambart | PopART | helen@zambart.org.zm |
| David Bonsall | University of Oxford | Sequencing lab | david.bonsall@bdi.ox.ac.uk |
| Rory Bowden | University of Oxford | Sequencing lab | bowden.r@wehi.edu.au |
| Vincent Calvez | Institut Pasteur | TasP trial | vincent.calvez@me.com |
| Max Essex | Harvard Botswana | Botswana studies | messex@hsph.harvard.edu |
| Sarah Fidler | PopART/Imperial College London | PopART | s.fidler@imperial.ac.uk |
| Christophe Fraser | University of Oxford | Principal Investigator PANGEA 2, Executive Committee and PopART Phylogenetics | christophe.fraser@bdi.ox.ac.uk |
| Kate Grabowski | Johns Hopkins University | Executive Committee and Rakai | mgrabows@jhu.edu |
| Tanya Golubchik | University of Oxford | Data manager | tanya.golubchik@sydney.edu.au |
| Ravindra Gupta | University of Cambridge | AHRI studies | rkg20@cam.ac.uk |
| Richard Hayes | PopART/LSHTM | PopART | Richard.Hayes@lshtm.ac.uk |
| Joshua Herbeck | University of Washington | Partners PrEP; Partners in Prevention | Josh.Herbeck@gatesfoundation.org |
| Joseph Kagaayi | Rakai Health Sciences Program | Rakai Health Sciences Program | jkagayi@rhsp.org |
| Pontiano Kaleebu | MRC/UVRI Uganda | MRC studies | pontiano.kaleebu@mrcuganda.org |
| Jairam Lingappa | University of Washington | Partners PrEP; Partners in Prevention | lingappa@uw.edu |
| Sikhulile Moyo | Botswana Harvard AIDS Institute Partnership | Botswana studies | sikhulilemoyo@gmail.com |
| Vladimir Novitsky | Harvard University | Botswana studies | VNovitsky@Lifespan.org |
| Thumbi Ndung’u | Africa Health Research Institute (AHRI) | AHRI studies | thumbi.ndungu@ahri.org |
| Deenan Pillay | University College London | Executive Committee | d.pillay@ucl.ac.uk |
| Thomas Quinn | Johns Hopkins University | Rakai Health Sciences Program | tquinn2@jhmi.edu |
| Andrew Rambaut | University of Edinburgh | Executive Committee | a.rambaut@ed.ac.uk |
| Oliver Ratmann | Imperial College London | Analysis | [oliver.ratmann@imperial.ac.uk](mailto:oliver.ratmann@imperial.ac.uk) |
| Janet Seeley | MRC/UVRI Uganda / LSHTM | MRC Uganda | Janet.Seeley@LSHTM.ac.uk |
| Deogratius Ssemwanga | MRC/UVRI Uganda | MRC studies | Deogratius.Ssemwanga@mrcuganda.org |
| Frank Tanser | Africa Health Research Institute | AHRI studies | ftanser@sun.ac.za |
| Maria Wawer^1^ | Johns Hopkins University | Rakai Health Sciences Program | mwawer1@jhu.edu |

It is the responsibility of each Steering Committee member to inform the PANGEA 2 Project Manager of any changes to the contact details.

# Appendix 2: PANGEA 1 Steering Committee

| Name | Institution | Study team representatives | Email address^3^ |
| --- | --- | --- | --- |
| Myron Cohen | University of North Carolina | -------- | myron_cohen@med.unc.edu |
| Tulio D’Oliveira | University of KwaZulu-Natal | -------- | [tuliodna@gmail.com](mailto:tuliodna@gmail.com) |
| Ann Dennis | University of North Carolina | -------- | ann_dennis@med.unc.edu |
| Max Essex | Harvard Botswana | Botswana studies | messex@hsph.harvard.edu |
| Sarah Fidler | PopART/Imperial College London | PopART Phylogenetics | s.fidler@imperial.ac.uk |
| Dan Frampton | University College London | -------- | [d.frampton@ucl.ac.uk](mailto:d.frampton@ucl.ac.uk) |
| Christophe Fraser | University of Oxford | PopART Phylogenetics | christophe.fraser@bdi.ox.ac.uk |
| Tanya Golubchik | University of Oxford |  | tanya.golubchik@sydney.edu.au |
| Richard Hayes | PopART/LSHTM | PopART Phylogenetics | Richard.Hayes@lshtm.ac.uk |
| Josh Herbeck | University of Washington | Partners PrEP; Partners in Prevention | Josh.Herbeck@gatesfoundation.org |
| Anne Hoppe | University College London | Project Manager PANGEA 1 / EARNEST | hoppe.anne@gmail.com |
| Pontiano Kaleebu | MRC/UVRI Uganda | MRC studies | pontiano.kaleebu@mrcuganda.org |
| Paul Kellam | Cambridge University | -------- | [paul.kellam@kymab.com](mailto:paul.kellam@kymab.com) |
| Cissy Kityo | EARNEST/JCRC Uganda | EARNEST | [ckityo@jcrc.org.ug](mailto:ckityo@jcrc.org.ug) |
| Andrew Leigh-Brown | University of Edinburgh | -------- | [A.Leigh-Brown@ed.ac.uk](mailto:A.Leigh-Brown@ed.ac.uk) |
| Jairam Lingappa | University of Washington | Partners PrEP; Partners in Prevention | lingappa@uw.edu |
| Vladimir Novitsky | Harvard University | Botswana studies | VNovitsky@Lifespan.org |
| Nick Paton | EARNEST / University of Singapore | EARNEST | [nick.paton@ucl.ac.uk](mailto:nick.paton@ucl.ac.uk) |
| Deenan Pillay | Africa Health Research Institute / University College London | Principal Investigator PANGEA 1 / Africa Health Research Institute studies | d.pillay@ucl.ac.uk |
| Tom Quinn | Johns Hopkins University | Rakai Health Sciences Program | tquinn2@jhmi.edu |
| Oliver Ratmann | Imperial College London | -------- | [oliver.ratmann@imperial.ac.uk](mailto:oliver.ratmann@imperial.ac.uk) |
| Deogratius Ssemwanga | MRC/UVRI Uganda | MRC studies | Deogratius.Ssemwanga@mrcuganda.org |
| Frank Tanser | Africa Health Research Institute | -------- | ftanser@sun.ac.za |
| Maria Wawer^1^ | Johns Hopkins University | Rakai Health Sciences Program | mwawer1@jhu.edu |
