# Supplementary material for: Limited emergence of resistance to integrase strand transfer inhibitors (INSTIs) in ART-experienced participants failing dolutegravir-based antiretroviral therapy: a cross-sectional analysis of a Northeast Nigerian cohort
Source: J Antimicrob Chemother. 2023 Jun 27;78(8):2000–7. doi: 10.1093/jac/dkad195 (PMC10393879; doi:10.1093/jac/dkad195)
Supplement: dkad195_Supplementary_Data [file dkad195_supplementary_data.zip › Supplementary Figures .docx]

**
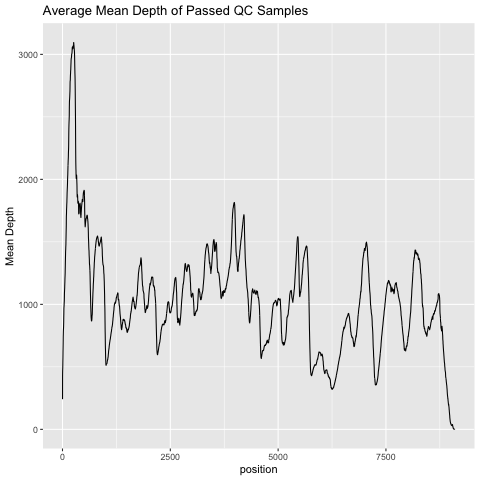
**

**Supplementary Figure 1:** Mean depth of BAM files utilised in analysis. The mean depth of the whole genomes exceeds 500 reads in all regions examined for drug resistance.

**
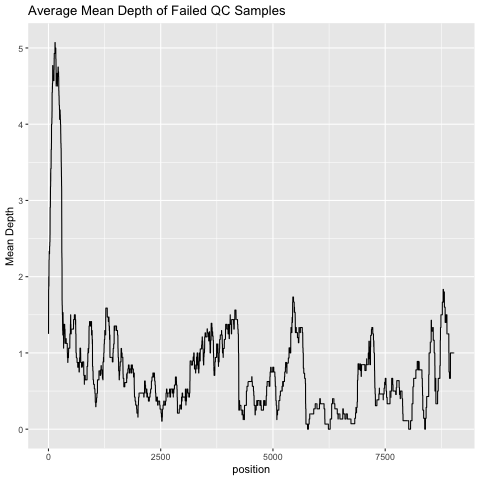
**

**Supplementary Figure 2:** Mean depth of BAM files excluded from the analysis.
